# “Am I really alive?”: Understanding the role of homophobia, biphobia and transphobia in young LGBT+ people’s suicidal distress

**DOI:** 10.1016/j.socscimed.2022.114860

**Published:** 2022-02-24

**Authors:** Hazel Marzetti, Lisa McDaid, Rory O’Connor

**Affiliations:** a School of Health in Social Science, University of Edinburgh, UK; b MRC/CSO Social and Public Health Sciences Unit, University of Glasgow, UK; c Institute for Social Science Research, University of Queensland Australia, Australia; d Institute of Health and Wellbeing, University of Glasgow, UK

**Keywords:** Suicide, LGBT, Sexuality, Transgender, Self-harm, Stigma, Youth, Qualitative

## Abstract

Suicide is the fourth leading cause of death amongst young people aged 15-29 globally and amongst this young population, lesbian, gay, bisexual and trans (LGBT+) young people have higher rates of suicidal thoughts and attempts than their cisgender (non-trans), heterosexual peers. However, despite well-established knowledge on the existence of this health inequality, in the UK there has been a paucity of research exploring why this disparity exists, and this is particularly the case in Scotland. This paper aims to address this gap, reporting on the first study specifically seeking to understand LGBT+ young people’s suicidal thoughts and attempts in Scotland. We used a qualitative methodology to explore how young people with lived experience of suicidal distress make sense of the relationship between homophobia, biphobia and transphobia, and suicidal thoughts and attempts. We undertook in-depth, narrative interviews with twenty-four LGBT+ people aged 16-24, and analysed them using reflexive thematic analysis. Drawing on this analysis, we argue that suicide can be understood as a *response* to stigma, discrimination and harassment, made possible by a cultural climate that positions LGBT+ people as *different* or *other*, reinforcing norms regarding gender conformity and sexuality. We suggest in turn, that this cultural climate provides fertile ground from which more explicit acts of homophobia, biphobia and transphobia, such as bullying and family rejection are able to grow. In response to this, LGBT+ young people could begin to experience senses of entrapment, rejection and isolation, to which suicidal thoughts and attempts can be understood as responses. Consequently, we propose that these stigma experiences must be taken seriously and tackled directly in order to reduce LGBT + suicide in the future.

## 1 Background

Suicide is a major public health concern: worldwide around 700,000 people die by suicide annually, and an estimated twenty times more **survive suicide attempts** (World Health Organization, 2021). **Globally, suicide is the fourth leading cause of death amongst young people aged 15-29 years** (World Health Organization, 2021). Over the past five decades, Scotland has consistently had a higher rate of deaths by suicide when compared to England & Wales (Dougall et al., 2017), with suicide named as the leading cause of death amongst people aged 5-19, and the second leading cause of death amongst people aged 20-34 in Scotland in 2019 (National Records of Scotland, 2019). Within this young population, global estimates suggest that LGBT+ young people (a term used to include lesbian, gay, bi and trans people, as well as anyone who defines their sexual, romantic or gender identity outside of the confines of simultaneous cisgender, heteroromantic, heterosexuality) are more likely than their cisgender (non-trans), heterosexual peers to think about and attempt suicide (Marshal et al., 2011; Surace et al., 2020), and this is **likely to be influenced by the social, legal and political contexts in which those young people live. However, very little research has focussed on the experiences of LGBT+ young people living in the UK**, and this is particularly the case for those living in Scotland.

Globally, explanations of the unequal burden of suicidal thoughts and attempts amongst LGBT+ young people have focused on experiences of homophobic, biphobic and transphobic (taken together queerphobic (Marzetti, 2018)) stigma, discrimination and harassment. This can be considered within the broader framing of negative LGBT+ health outcomes (both physical and mental health), in which expecting, experiencing, and internalising queerphobia, along with concealing one’s LGBT+ identity to avoid it, is termed ‘minority stress’ (Meyer, 2003). Amongst young LGBT+ people specifically, high levels of bullying and victimisation (Myers et al., 2020; Williams et al., 2021); family rejection or a lack of family support with regard to one’s LGBT+ identity (Bouris et al., 2010; Schnarrs et al., 2019); stress related to coming out (Rivers et al., 2018); and more generally living in a community that is negative about LGBT+ people (Meyer et al., 2019) may enact these minority stresses. This can then interact with other stresses that young people experience, such as mental health problems, difficulties at school, and experiences of abuse, further negatively impacting their mental health (Rimes et al., 2018; Rivers et al., 2018).

LGBT+ youth suicide research in the UK has primarily used quantitative survey methods (Oginni et al., 2018; Rimes et al., 2018, 2019), with little work qualitatively exploring suicidal distress from the perspectives of those young LGBT+ people who experience it (McDermott and Roen, 2016). As a result, although experiences of stigma, discrimination and harassment are consistently positioned as stresses associated with suicidal thoughts and attempts, the ways in which these are understood by LGBT+ young people, ‘getting under the skin’ and contributing to suicidal distress, is less clear (Hatzenbuehler, 2009). Although this is now beginning to be addressed through an emerging literature on the experiences of LGBT+ young people in England and Wales (McDermott and Roen, 2016; Nodin et al., 2015; Rivers et al., 2018), there has been little exploration of the lived experiences of suicide amongst LGBT+ young people in Scotland.

One possible interpretation of queer youths’ suicidal thoughts and attempts has been offered by McDermott and Roen’s (2016) qualitative work on queer youth suicide in England and Wales, which suggested that queer youths can come to perceive themselves as failing by multiple normative, neo-liberal standards. They argue that in embodying queer genders and queer desires, and expressing distressed, at times suicidal, emotions, queer youths persist in having feelings and experiences that society expects them to ‘grow out of’ or ‘get over’ (McDermott and Roen, 2016). Crucial to this argument are theories of normativity: centrally, cisnormativity (sometimes termed cisgenderism) and heteronormativity. It has been suggested that explicit acts of homophobic hatred have, over time, reduced; however heteronormativity, positioning heterosexuality as ‘normal’ and desirable, has persisted (Cover, 2012). A similar argument can be made about the persistence of cis-normativity which positions being cisgender and gender conforming as ‘normal’ and desirable (Ansara and Hegarty, 2012; Bauer et al., 2009). The term cis-heteronormativity has been used to combine these normative pressures and describe times at which it is not possible to disentangle them from one another (Marzetti, 2018).

To further explore this concept, we found Sara Ahmed’s theory of coming out as disorientation a useful conceptual frame (2006). Ahmed argues that one’s sexual orientation is not simply an orientation of desire, but an orientation within society. Building on Adrienne Rich’s (1980) work, Ahmed suggests that through constant, repeated exposure, heterosexuality become normalised and expected. Within this context, living heterosexually is quite literally a life that goes with the expected flow; and in contrast, life as an LGBT+ person therefore is a process of disrupting those expectations. To extend this idea, heteronormativity can perhaps be conceptualised as a tide: if you are swimming in the sea and the tide is *with* you, you may not notice it silently helping you move forward or if you do it is with recognition that it is helping you to reach your destination. If, however, you are swimming *against* the tide its resistance is fully felt; it is unable to be ignored.

It was in response to the perceived transgressions of society’s normative standards that McDermott and Roen (2016) suggest queer youths can experience societal sanctioning through the enactment of stigma from those around them. In turn, this can result in queer youths experiencing mutually reinforcing senses of isolation and shame, from which suicide may present as the most visible or accessible escape. This argument therefore presents a further question: how does suicide become the most visible or accessible form of escape from queerphobia and cis-heteronormativity for LGBT+ youths in distress? In this paper, we explore the ways in which LGBT+ young people themselves make sense of the relationship between their LGBT+ identity and suicidal distress; reporting on the findings of first qualitative exploration of LGBT+ young people’s suicidal thoughts and attempts in Scotland. To begin, we consider the ways in which pressures to conform to norms of sexual orientation, gender identity and gender expression were reported to be enforced by participants’ peers, families, and wider society. We then go on to discuss the ways in which participants understood suicidal thoughts and attempts as *responses* to this, conceptualising suicide as both a route for escape and a tool for questioning one’s value to others.

## 2 Methods

This paper draws on in-depth, narrative interviews with twenty-four LGBT+ people aged 16-24 with lived experiences of either suicidal thoughts or attempting suicide, living in Scotland.

## 3 Recruitment

Participants were recruited between May and October 2019 using advertisements distributed through LGBT+ community events; partner organisations and their events; social media (Facebook, Twitter and Instagram); and through research participants proactively promoting the research in their own networks. Our inclusion criteria required participants to: be aged 16-24; live in Scotland; have lived experience of either suicidal thoughts or a suicide attempt; and self-identify as LGBT+. Although participating in suicide research has not been shown to increase participants’ suicide risk (Blades et al., 2018), it was essential that participants’ wellbeing was prioritised at every stage of the research, taking proactive steps to mitigate risks. In advance of the research interview, all participants took part in a phone or video call, part of which focussed on the support structures they would feel comfortable accessing if they found participating in the research distressing. Subsequent to the interviews, all participants received an information sheet with contact details and opening times for organisations that provide mental health, suicide or LGBT+ specific support. All participants were also offered an optional follow-up phone call to discuss the research. Ethical approval for the study was granted through the authors’ university’s research ethics committee.

## 4 Sample

All 24 participants in this study had lived experience of suicidal thoughts. Ten participants had attempted suicide, all more than once. Participants were aged between 16 and 24 years (with an average age of 19.6) and were from a range of urban and rural locations across Scotland. Seven participants described their gender as men or male (six trans and one cis). Eleven participants described themselves as women or female (all cis). Six participants used terms outside of the binary of man/woman to describe their gender identity: two participants were non-binary; one was trans non-binary; one was a female tomboy; one was a transgender demiboi; and one was a non-binary trans woman. Ten participants were trans. Participants were invited to describe their sexual and romantic orientation using as many terms as they felt were appropriate. Eighteen people used non-monosexual terms: pansexual (seven); bisexual (six); queer (three); bi (two); biromantic (one), whilst seven people used monosexual terms: lesbian (three); gay (three); homosexual (one). One participant described themselves as ace, one as asexual, and one as aromantic.

## 5 Interview methods

**Fig. 1:**
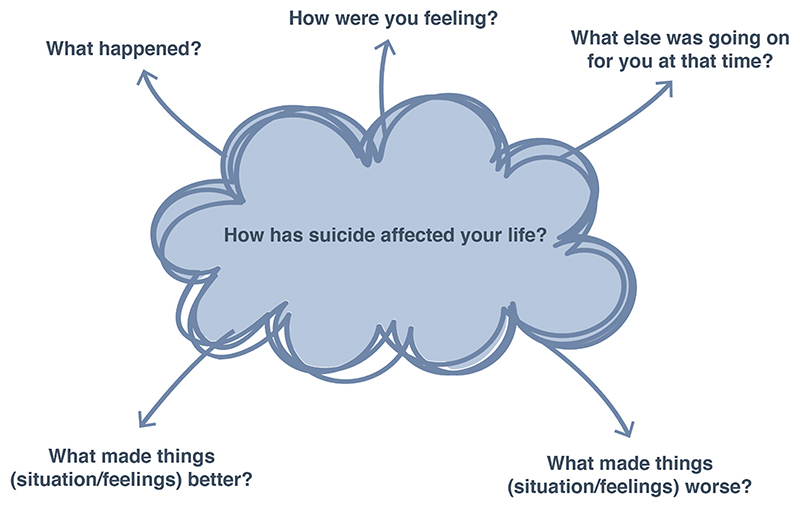
Interview schedule - paper based resource.

**Fig. 2:**
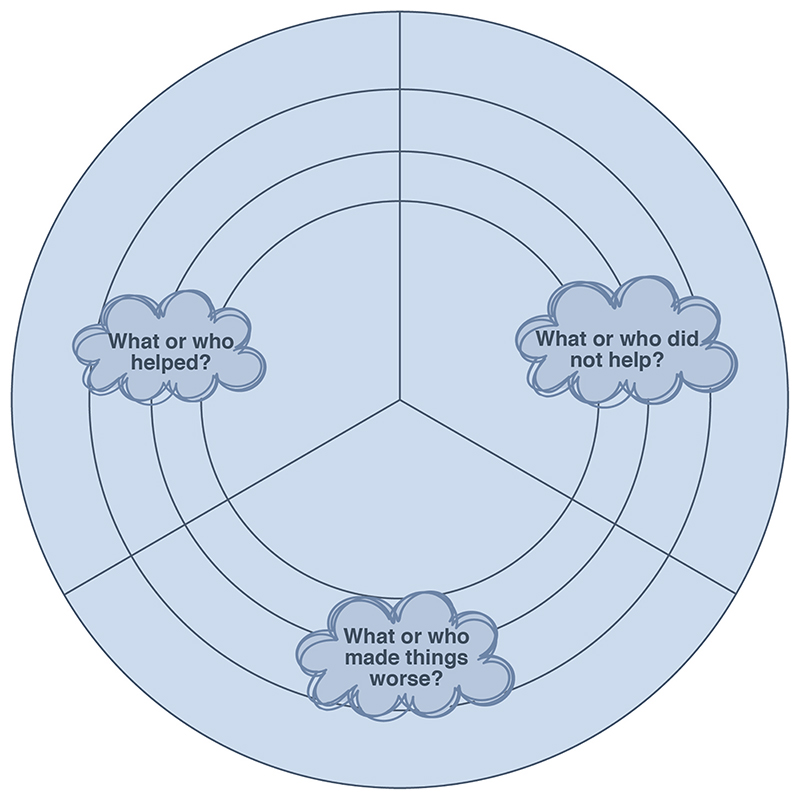
Reflection wheel - paper based resource.

Given the sensitivities of the topic, interviews were designed to allow participants space, time and privacy in which to share their stories (Fontana and Fey, 2003). We used loosely structured narrative interviews to provide a small number of clearly defined yet open questions. The openness of the questions aimed to support participants to steer the conversation in directions they deemed important (Burgess-Proctor, 2015; Riessman, 1987). This was complemented by structured visual representations of the interview questions (Figs. 1 and 2), designed to avoid the ‘interpretative problem’, wherein vague interview questions may result in over interpretation where participants try to frame their answers to the question that they think their interviewer is trying (but failing) to ask (Silverman, 2001). The presence of printed interview questions afforded participants certainty about what they would be asked, whilst also providing a focus point that participants could draw or write on if they did not want to engage directly with the interviewer. This was positively remarked upon by one participant who found eye contact with others particularly difficult.

Interview questions and the corresponding paper resources were designed and refined in dialogue with partners from LGBT+ and youth charitable organisations, and then further refined through three pilot interviews. Throughout the interview dialogue was further facilitated by actively listening and using silence, echo probes (repeating back a phrase the participants has used), neutral probes (encouraging noises), and follow on questions (Hesse-Biber, 2011). With participants’ consent, all interviews were audio recorded, transcribed in full by a professional transcription company, anonymised and participants were assigned pseudonyms. All participants were offered the opportunity to select their own pseudonyms, as has been described as good practice particularly when working with trans participants (Vincent, 2018), and were explicitly asked for their pronouns.

## 6 Analysis

The transcripts were checked for accuracy against the audio recording and read in full to re-familiarise the first author with the data. After the initial re-reading, summaries of each participant’s stories were written by the first author as an individual, reflective task. Through this process elements of participants’ stories considered particularly analytically salient were drawn out and were considered for points of convergence and divergence in relation to other participants’ stories as well as the research literature (Braun and Clarke, 2006, 2020). To accompany this, reflections on the narrative composition of participants’ stories, considering *how* and *why* accounts were constructed (Josselson, 2012; Whitaker and Atkinson, 2019), added an additional layer of analysis. A process of noting was used to capture initial responses to the interview, often through written questions, as well as documenting early ideas for codes, written in the margins of the printed transcripts.

Following the initial exploratory analysis, the first author coded each interview individually, constructing a proliferation of codes that were then refined through multiple readings of the data, and the codes grouped by centrally organising themes. This was supported by reflective journaling around Sustein and Chiseri-Strater’s prompts ‘What surprised me? What intrigued me? What disturbed me?’ (2012; pp.115), which helped explicitly identify and then consider how personal assumptions, positionality and values influenced analysis. Following this, the first author undertook a process of descriptive writing about the themes generated. The descriptive writing was then shared with the second and third authors and the first author’s other PhD supervisor who asked a range of critically engaging questions. This was explicitly not a process of ‘checking’ the codes generated (Braun and Clarke, 2020), but was instead a process of critical engagement and discussion in order to deepen and develop analysis (Barbour, 2002), and was used to further refine the themes.

## 7 Findings

The findings that follow focus on how participants’ made sense of queerphobia in relation to their stories of suicidal thoughts and attempts. Firstly, we explore LGBT+ specific factors considered contributors to suicidal distress, discussing how societal norms can provide fertile ground on which both queerphobic bullying and family rejection of LGBT+ identities are able to grow. Secondly, we consider the ways in which LGBT+ youths’ suicidal thoughts and attempts can be conceptualised *as responses*: both as potential means of escape from stigma, discrimination and harassment, and as a way of embodying the rejection, isolation and othering they felt, questioning their value to others. All quotes presented in this article are illustrative of the broader themes constructed.

## 8 Queerphobia as inescapably everyday

### 8.1 Cis-heteronormative community climates

Participants described how everyday comments, questions and looks could serve to establish and re-establish cis-heteronormativity on a day-to-day basis.

Lynsey (21; she/her): [town’s] the type of place where if you deviate from the norm, the norm being like what a typical idea of what a man and a woman is, you get kind of weird looks. Like when I go home now, people stare at me on the street, because obviously I walk about with a bald head, […] when I say small-town mentality, it was very, like, if you’re different, you were like … it was not a pleasant place to live.

In this quote, Lynsey discusses how her gender non-conformity was responded to in her hometown. This provides one example of how queer bodies can be viewed by those holding cis-heteronormative expectations as a disruption to this societal orientation, which is then sanctioned through subtle, yet stigmatising, gestures such as the “weird looks” Lynsey describes. These subtle expressions of cis-heteronormative stigma were also echoed by other participants, describing their home-towns as “inherently narrow-minded places”, “quite judgemental”, and as having “ingrained sexism, and racism, and homophobia”.

Whilst for some participants in this study, the cis-heteronormative community climate was established through in-person interactions such as the one Lynsey described. For others, this was also contributed to by online or media interactions that were negative about LGBT+ people, with one participant, Stuart, describing how witnessing online transphobia had “reinforc[ed] my personal need to stay stealth [a term used to describe a trans person who is not out as trans] in most things”. Taken together, these everyday interactions shaped how participants expected to be treated. It was against this backdrop of expected non-acceptance that participants described experiencing all other everyday experiences and challenges:

Yasmin (19; she/her): For LGBT+ young people specifically, just societally, if you have a feeling, especially when you’re young that you’re not going to be accepted and it’s going to be harder for you to sort of move through the world because of your identity that brings a real feeling of hopelessness.

In this quote, Yasmin expresses the difficulties of navigating everyday life whilst expecting and experiencing a deep sense of rejection for one’s LGBT+ identity. Therefore, whilst cis-heteronormativity was not cited by participants as a direct catalyst for suicidal thoughts and attempts, it appeared to cultivate a fertile ground on which other, more direct contributors, were able to grow.

### 8.2 Queerphobic bullying

The majority of participants reported bullying throughout their education. Many participants described this bullying as targeting their perceived gender non-conformity, which they believed was often interpreted as evidence of a non-heterosexual orientation. Queerphobic bullying therefore served to extend and amplify the cis-heteronormative community climates in which participants lived, serving as a tool through which they were sanctioned for transgressing these norms.

Ayla (18; she/her): one of the slurs the guy used was “genderless” because I hung out with boys as much as I did with girls and like that … and I do not really care that much about my physical appearance, to be honest, when I was like younger but I wasn’t again an exact tomboy because I had [redacted hobby perceived by participant to be typically feminine], so I was like in the middle thing, like people were like, “what are you?”.

In this quote, Ayla’s bullies appeared to target her gender non-conformity as a way to more fundamentally question, and in turn undermine, her personhood through the question “what are you?”. Ayla described how these attacks on her physical appearance had a long lasting impact on her relationship with herself:

Ayla (18; she/her): some of the things they said on a regular basis was like, “you are the ugliest person in the world, like no-one will ever love you”, and things like that. Like once I feel like that becomes a thing you hear all the time you believe in it. It’s like, although after I finished secondary school, although I didn’t hear from them again for a long time, it was like they left but I kind of created this bully that was inside me and like even after losing my contact with them I realised I had the same pressure on me, myself now, like “why are you like this? You are so ugly. You’re never enough!”, and things like that.

For Ayla, as was the case for other participants, the internalisation of bullies’ voices meant that although she moved away from her bullies, they had a long-term impact on her self-esteem and self-compassion, and therefore were central to her own understanding of her on-going suicidal distress.

As a tool for managing the distress arising from bullying, some participants appeared to minimise and normalise queerphobia as part of the daily discourse of LGBT+ lives.

Andrew (20; he/him): It’s just your kind of playground kind of gay bullying, kind of gay bashing, if you like […] Just the usual, it was just like being intimidated, I think I was beaten up a few times, I’ve been followed home a few times, only run of the mill [laughter]; which is really sad that I say that, but I think it does ring true, it’s your kind of run of the mill gay sob story almost. But yeah, that was just really isolating in school.

Coming to understand one’s self as a victim can sometimes be accompanied by feelings of shame (Ridge et al., 2020). Responding to this, minimising and normalising bullying has been discussed as a method of resisting shame and victimhood, positioning one’s self as mature, strong and proud (McDermott et al., 2008; McDermott and Roen, 2016; Scourfield et al., 2008). This is particularly the case in an LGBT+ context, where expressing shame can be viewed as an absence of pride in one’s LGBT+ identity, where pride is almost expected amongst LGBT+ young people (McDermott et al., 2008). In this quote, although Andrew gives an account of his social isolation through bullying, he simultaneously appears to minimise it, narratively positioning this experience of isolation and victimisation as part of a ‘normal’ gay experience. In doing so, he seems to realign his sense of belonging with a new sense of gay normality, and this minimisation is further reinforced through the subtle repetition of “just” throughout the quote.

### 8.3 Coming out and family responses

Navigating an anticipated or actualised negative response to coming out was cited by some as making suicidal distress worse and was described by participants in a manner that suggested enormous emotional demands. In anticipation of negative responses, some participants chose to come out to their families at a distance through letters or videos, whilst others had avoided coming out to them altogether, and two participants described trying to change their sexual orientation through prayer.

Eilidh (21; she/her): I used to like pray to God, don’t make me gay, I’ll be like such a good Christian […] I was just very like I’ll do all these things for you. And that never worked. And I’d be like I’ll google at-home conversion therapy because I was like I don’t want this.

In this study, concerns about negative family reactions were possibly amplified, as participants described beginning thinking about suicide aged 14 or younger. As a result, many were very often materially dependent on their families for financial support, shelter, emotional support and nourishment that could be withdrawn when in conflict.

Archer (17; he/him/they/them): [after coming out as trans] my granny also started getting on my mum’s case, telling her to chuck me out and stuff and being like, “show her the door, that’ll give her a scare”, and stuff.

Some participants expressed a sense with which they felt pressure to carefully balance parental desires with their own life satisfaction with regard to sexual orientation and gender identity.

Stromberge (19; he/him): Even if I tried to calmly discuss stuff with my mum, it would end up in an argument […] she said it was permanent stuff [related to his transition] that she was worried about, so I went, okay, let’s have a think. And I talked about, you know, I went, well, clothes, and hair, and name, and pronouns aren’t permanent. And she just absolutely, you know, threw that out and was like, no.

This quote illustrates a broader sentiment expressed by many participants, in which their families either had expressed a lack of acceptance for their LGBT+ identity or had found the participant’s coming out extremely emotionally distressing. Consequently, as these emotional reactions were interpreted as responses to the young person’s coming out, some participants understood it as their responsibility to make the situation better. Within this, participants appeared to be attempting to balance their queer existence with their families’ queerphobia, undertaking the perhaps near impossible task of living queerly enough to be comfortable themselves, whilst concealing enough of their identity to avoid prompting family conflict. As a result, this balancing process seemed to significantly limit the ways in which young people could exist comfortably.

In many participants’ accounts, familial conflict centring on the rejection of their LGBT+ identity, appeared to be perceived by both families and participants as, at least in the short-term, immutable and irresolvable.

Stromberge (19; he/him): I always think you know what, if I work hard, and I put the effort in it’ll work out. Whereas, this [conflict with his mother around transition] was something where I couldn’t even figure out how to work hard, and put the effort in, not to mention, do that and get it to work out you know.

This conflict appeared to be somewhat rooted in differing perceptions of the ontological permanence and significance of participants’ LGBT+ identities. As whilst families may have understood their rejection of an individual’s LGBT+ identity as a rejection of one part of them, in a manner that suggests that this could be separated from other elements of their identity. For participants, this rejection could be understood as a rejection of their personhood as a whole; without their LGBT+ identity they simply did not exist in a manner recognisable to themselves.

## 9 Understanding suicide as a response

### 9.1 Queer entrapment and suicide as escape

Participants’ experiences at home, at school, and in wider society, meant that the exploration and articulation of their personal identity, particularly with regard to their LGBT+ identity, was undertaken within a context of sustained rejection in at least one, if not many, areas of their life. Given these pressures, some participants expressed difficulties envisaging the future and described a sense of what we have termed ‘queer entrapment’, in which queerphobic conflict about their LGBT+ identity was perceived to be irresolvable and from which suicide was seen as an escape.

Lily (24; she/her): there have been times when I’ve just been like, oh, if I just ended my life it would just stop everything […] No one would have to deal with it, no one would have to be like, “oh, we’ve got a gay daughter” - no one would have to deal with it, it would just stop all the problems. I felt like that was the only way out of it all was just to like disappear.

In this quote, Lily describes her response to a difficult and on-going conflict with her parents related to her sexuality, in which at one point Lily described her father accusing her of “destroying the family” when she came out as a lesbian. Within this account, Lily presents suicide as an escape for herself, but further to this, in describing her existence as something that her family had to ‘deal with’, she also appears to position herself as a burden to her family and conceptualise her suicide as a way of ending this burdensomeness upon them.

For some participants, feelings of queer entrapment related directly to a sense of impossibility regarding their futures as LGBT+ people. Amongst trans participants, delays and difficulties medically transitioning were cited by some as contributing to feeling ‘stuck’ and as if life was ‘entirely pointless’.

Lewis (21; he/him): transitioning felt like a different dimension, like it wasn’t possible, like I would never be able to be free as such, kind of felt as if it was a cage that I couldn’t get out of. So, kind of the last resort was … the only way to escape it was to die.

Concerns about the impossibility of the future were not limited to trans participants. Throughout his interview Euan, who uniquely in this study considered himself not to be ‘out’ as a cisgender, gay man, repeatedly described the ways in which he felt trapped and torn by his own internalised homophobic shame and in which he consciously tried to embody what he perceived to be heteronormative, masculine gender norms.

Euan (21; he/him): I’m not ashamed but I am ashamed, but I don’t want people to think I’m gay but I want people to think I’m gay […] I put down on what I want for the future as coming out, and it’s like I don’t think it’s going to happen, I want for it to happen, that’s what I want for the future, I want to be that perfect image of myself, fully accepting myself, fully happy, but trying to live it? I can imagine it, but I can’t live it. It’s like when I try to go towards it, it feels different than thinking it in my head, and it’s like it’s so much effort, it’s so much work and it’s so … I don’t know how people have the strength to stay out.

Whenever Euan had tried to come out he had found himself met with shocked responses or invasive questions, these reactions were then reinforced for him by heteronormative expectations that he heard expressed around him, for example being told ‘guys bring your girls’ to a work event. Taken together, although he expressed his desire to come out and live openly as a gay man, he was simultaneously trapped within his internalised homophobic shame and therefore felt unable to do so. Consequently, he described feeling that eventually he would ‘do’ (come out) or ‘die’ (by suicide).

### 9.2 Suicide as questioning existence

Whilst for some, suicide provided an escape from an intolerable situation, for others suicide confronted the sense of existential rejection they experienced from others. In this sense, they internalised, embodied and enacted this rejection on their bodies through suicidal and self-harming practices.

Lewis (21; he/him): The first time I felt suicidal must have been about thirteen, fourteen, didn’t really know what it was, to be honest, didn’t know what suicide was, I just was like, I don’t really feel anything, so like self-harm was a way to feel anything, like to feel that I was actually still alive, like because of just constant numbness, you’re like, am I really alive, can I feel things? Because I don’t think I can feel anything, so even just pain is like, okay, I’m still alive, seeing blood, still bleeding, my heart’s still working, still here, but then that becomes into a habit, and it’s like the only way to feel. Don’t want to do this. And because like nobody had really noticed, and I was like, well, nobody would really notice if I wasn’t here then.

Here, Lewis’ account presents his self-harm practices as being used as an embodied confirmation of his existence, disrupting disassociation and allowing him to feel something (anything) when feeling otherwise numb.

The understanding of self-harm or suicide as an embodied practice of existential questioning was echoed by other participants. In turn, these practices could form part of a dialogue in which both self-harm and suicide were positioned as a call that invited a response, exemplified in the variations of Lewis’ refrain “nobody really noticed” in the quote above. I interpreted this questioning not simply as seeking to answer questions about their own existence, but further as questioning whether their existence mattered *to others*.

Andrew (20; he/him): It was something that would linger in my mind, the kind of existential questions, like who’s going to notice, and what else was it? What difference is it going to make, those kinds of existential questions.

Using this interpretation, this questioning cannot be considered as solely situated in individuals’ psychologies. Instead, suicidal distress should be understood as situated in the interactions *between* the suicidal individual, the context in which they live, and the interpersonal relationships that they have.

Sophie (18; she/her): At one point I would have people at school, my dad, and my brother, all at the same time, with different intents, telling me, you’re disgusting, it’s fucking wrong. And if someone tells you something enough, you start to believe it.

When a young person is rejected, isolated and told they are a burden, such as in the manner reported in Sophie’s quote, their diminishing self-esteem and self-compassion must not be purely seen as a result of their perceptions. Instead, it should be understood, at least in part, as *responding to* these negative interactions. This is not to say that suicide should be seen as an automatic or immediate response to queerphobia and cis-heteronormativity. However, it is to argue that where participants felt that they were not cared about and that their life did not, or might not, matter to those they loved, suicide could be understood as an embodied enactment of this rejection on the self.

## 10 Discussion

Consistent with Minority Stress Theory, participants in this study explored the ways in which expecting and experiencing queerphobia was an inescapably everyday phenomena, contributing to participants’ feelings of rejection and isolation. Previous research has identified how feeling accepted where one lives and experiencing a community climate that is positive for LGBT+ young people has been considered protective against suicidal distress, whilst not feeling accepted is thought to contribute to suicidal thoughts and attempts (Hatzenbuehler, 2011; Meyer et al., 2019; Rimes et al., 2018). However, understanding why a community feels un/safe beyond direct experiences of harassment can be difficult due to the subtle and normalised nature of the practices creating this community climate (Cover, 2012; Goffman, 1963; Link and Phelan, 2014; McDermott and Roen, 2016).

In this study, we were able to tease out how everyday comments, questions and looks served to remind LGBT+ young people of cis-heteronormative expectations and highlight their transgressions of those expectations. Cis-heteronormativity was often not upheld with malicious intent, but instead through everyday innocuous practices such as the presumption of a different-gender partner, which meant that it was not easily understood as a contributor to suicidal distress. However, it was exactly these everyday normative practices, establishing being cisgender and being heterosexual as not only ‘normal’ but also desirable, that created a community climate in which more overt and malicious acts of queerphobia were made possible and in which living an authentic LGBT+ life safely could become unimaginable.

These findings build on the work of Cover (2012) and McDermott and Roen (2016), who have argued that the pressure to conform with normative standards of maturity, emotional regulation and sexuality can have a profound impact on queer youths’ suicidal distress. In looking at the specific presentations of cis-heteronormativity, we have been able to explore in detail how this cultural climate cultivates a fertile ground in which both queerphobia and suicidal distress is able to grow. Indeed, it was against this backdrop of cis-heteronormativity that expectations and experiences of queerphobia were formed, resulting in feelings of isolation, rejection, being unwanted and not belonging in both schools and homes that were pervasive across the study. Participants described difficulties before, during and after coming out, which echoes previous research that has identified initial instances of coming out as a critical time for emotional and suicidal distress amongst LGBT+ young people (Rivers et al., 2018; Skerrett et al., 2017). During this period of conflict, some participants found themselves trying to exist in what has been described by McDermott and Roen (2016) as a “constrained space” (pp.114), in which the young person tried to find a way to exist that both allowed them to explore their sexual orientation or gender identity authentically, but that was simultaneously viewed as acceptable enough to be without social sanction.

Fundamentally underlying the relationship between cis-heteronormativity, queerphobia and suicidal distress appeared to be an ontological questioning of the nature of LGBT+ people’s existence. Butler (2004) has argued that the gendered embodiment of LGBT+ people can be so fundamental to one’s personhood that recognition of gender presentation and expression constitutes an essential part of recognition *as people*. This embodiment is not simply a question of what one *does*, it is what one *is* and how one is recognised *as human*. Without recognition *as LGBT*+ *young people*, participants in this study could lack recognition *as people*. It was then within this context of existential rejection that participants could experience a sense of what we have termed queer entrapment, from which life, for some, could become unliveable. This was then further compounded for some trans participants in the study, who could experience additional feelings of entrapment specifically related to medical transitions, where delays or difficulties were experienced accessing gender-affirming medical treatments. This is consistent with existing research which has found that trying to access medical transition can be frustrating and have negative effects on individuals’ mental health (Bailey et al., 2014; Carlile, 2019; Dhejne et al., 2016; Ellis et al., 2014).

Within this context, suicidal distress can be understood not as solely situated in the cis-heteronormative community climate, nor the queerphobia found in interpersonal relationships, nor in individuals’ psychological states; but as a response located in the interactional spaces *between* them. Both cis-heteronormativity and queerphobia worked as a call to action to conform to norms regarding both sexual orientation and gender identity, thus inviting a response. Using Butler’s theory of recognition however, this question can be interpreted not simply asking them to *do* something differently in conforming to these norms; but instead to *be something different*, to fundamentally transform who they are *as humans*.

In response to this call, suicide was framed as an option both for escape and as an embodied practice of internalising, enacting and thus questioning the rejection that they had experienced externally. Although feelings of entrapment (O’Connor and Kirtley, 2018), leading to tunnel vision in which suicide is positioned as the *only* option for escape (Harris et al., 2010) has been discussed in suicide research, in this article we have explored why this may be more widely experienced by LGBT+ young people. For some participants feelings of queer entrapment, exacerbated by participants’ material dependence on their parents, could mean that conflict felt both inescapable and irresolvable and that therefore suicide could be viewed by participants as their only option for escape.

For others however, suicide and self-harm were positioned as part of an on-going interaction between the participant, their community climate and interpersonal relationships. This builds on work examining the interactive and communicative function of self-harm, in which Steggals et al. (2020) have argued that where the limitations of language are felt in expressing distress, self-harm can be used both to communicate and authenticate one’s feelings by inviting recognition of them by others. In this study, suicide appeared for some to respond to the existential rejection they faced from those around them, through the internalisation of that rejection. This appeared to somewhat demonstrate the ways in which a rejection of their sexual orientation or gender identity was a rejection of participants’ whole existence; internalising, embodying and enacting this rejection on the participants’ own bodies. Through these practices, participants sought an embodied confirmation of their existence and the ways in which that existence did or did not *matter* to those around them.

In exploring the ways in which LGBT+ young people themselves made sense of the relationship between their LGBT+ identity and suicidal distress, we found that both queerphobia and cis-heteronormativity were, in many ways, central. Considering this perhaps begs a further question: what can be done? In answering this, we can turn towards suicide prevention strategies, which are considered integral to national suicide prevention work (World Health Organization, 2021). Although suicide prevention strategies could offer a somewhat unique opportunity to holistically consider suicide away from the confines of a clinical setting (Yip, 2005), such policies often focus on individualistic, medicalised solutions that fail to consider the potential to intervene in the broader social contexts in which suicide happens (Fitzpatrick et al., 2015; Marzetti et al., 2022).

When we asked participants in this study what they believed would help reduce LGBT+ youth suicide in the future, it was exactly these social contributors to suicidal distress that they focussed on; suggesting tackling queerphobia at its roots. To do so, they proposed that LGBT+ people should be proactively and sensitively included on the school curriculum; represented in popular culture (TV, film, books, etc) in ways that did not promote stigma and stereotypes; and that mental health services should have LGBT+ awareness, but where this was not possible, should be able to refer to services that did. Therefore, whilst this might not feel like a radical solution, extending suicide prevention beyond direct mental health care for those experiencing distress, into the social structures that, at least in part, contribute to it would in fact enact a radical reshaping of conventional suicide prevention efforts.

## 11 Reflections and limitations

Firstly, although this study deliberately aimed for breadth and openness in the interview schedule (which focussed on the question ‘how has suicide affected your life?’), the majority of participants described extensively the ways in which cis-heteronormativity and queerphobia had impacted upon their lives. In considering this focus, we must acknowledge interviews *as accounts*. By this we mean that interviews are co-produced by interviewers and interviewees in their interactions, and therefore their expectations and perceptions of each other shape the narrative of the interview (Whitaker and Atkinson, 2019). It is possible that participants in this study focussed on their experiences of queerphobia and cis-heteronormativity because they knew that the study focussed on the experiences of LGBT+ young people and either perceived us as wanting to hear about queerphobia and cis-heteronormativity or believed that these were experiences that would be shared by most of the participants in the study. Secondly, although only people aged over 16 were included in this study, all participants reported beginning to experience suicidal distress aged 14 or younger. Therefore, future research should seek to work with LGBT+ people aged 16 or younger in order to better understand their experiences and the targeted support that would be most effective for this age group. Finally, given participants’ wide range of LGBT+ identities in this study, in addition to diversity of other characteristics (including, but not limited to, ethnicity, geographical location, disability, education level, and class), it was not possible to draw comparisons within the sample. In future research, it might be interesting to consider differences in experiences and needs at the intersections of a range of participant identities.

## 12 Conclusion

To the best of our knowledge, our paper reports on the first qualitative study aiming to understand LGBT+ young people’s suicidal thoughts and attempts in Scotland. In this paper, we explored the ways in which LGBT+ young people themselves make sense of the relationship between their LGBT+ identity and suicidal distress. We found that despite claims that Scotland is the best place in the UK to be LGBTI (Scottish Government, 2017), consonant with findings of research in the other UK nations (McDermott and Roen, 2016; Rivers et al., 2018), cis-heteronormativity and queerphobia were described as central contributors to suicidal distress. In exploring LGBT+ young people’s own ways of making sense of the relationship between cis-heteronormativity, queerphobia and suicide, we were able to examine the ways in which everyday, seemingly mundane, practices created a community climate in which both queerphobia and suicidal distress was able to grow.

It was within this context that participants articulated a sense of queer entrapment, in which they were rejected, isolated and consequently some lacked a safe space in which to exist *as LGBT*+ *people*. As a result, we proposed that suicide was constructed as a *response*: for some participants, as an escape from the sense of queer entrapment this engendered; for others, as a tool through which they embodied the rejection they experienced to question their value to others. Crucial to this argument is the notion that family rejection of LGBT+ identity and queerphobic bullying in educational institutions are not understood as an interruption to an otherwise accepting and affirming status quo. Instead we argue that they are made possible, in part, because they are continuous with and extensions of the pervasive cis-heteronormative cultural climate, and it is this cultural climate that needs to be disrupted in order to prevent LGBT + youth suicide in the future.

## Data Availability

The data are not publicly available due to ethical restrictions.
